# The architecture of creditions: Openness and otherness

**DOI:** 10.3389/fpsyg.2022.926489

**Published:** 2022-09-21

**Authors:** Oliver Davies

**Affiliations:** ^1^King’s College London, London, United Kingdom; ^2^LSRI Research Institute at Campion Hall, University of Oxford, Oxford, United Kingdom; ^3^Wolfson College, University of Oxford, Oxford, United Kingdom

**Keywords:** community, otherness, creditions, interdisciplinarity, integration, freedom, Franciscans, belonging

## Abstract

“Creditions” are an important new idea within our contemporary understanding of the human. They potentially represent the unity of both humanistic and scientific ways of modeling the human. As such, “creditions” offer a bridge between current thinking in science and the humanities and the development of a more powerfully integrated interdisciplinary hermeneutic. It is argued in this article that the questions posed by “creditions” (as developed by Rüdiger Seitz and Hans-Ferdinand Angel) cannot be resolved through reduction but rather only through cohesive systematization. In contrast with coherence in conventional science, “credition-centered” thinking finds expression in systemic ways. The complex humanity of the reflective subject resists reduction; and calls to be analyzed in terms of sociality, the identification of “otherness” and interactive engagement. In this context then a thinking which is attuned to complexity and to otherness has an important place in the expression of the social subject as a complex and relational self, in today’s world. These are not however social realities as we find them either in large-scale social schemata, or indeed in the intimacy of the face to face. Rather credition-centered learning falls between these two categories and is best described as “the productive knowledge of community,” where community is generated by productive enhancement and the embrace of otherness over time.

## Introduction: Contribution to an architecture of creditions

The concept of credition is an important new idea within our contemporary understanding of the human (Angel et al., 2017; see also Seitz and Angel, 2020). Creditions potentially represent the unity of both humanistic and scientific ways of modeling the human. As such, the concept of credition offers a bridge between current thinking in science and the humanities and the development of a more powerfully integrated interdisciplinary hermeneutic. The complex humanity of the reflective subject resists reduction; and calls to be analyzed in terms of sociality, the identification of “otherness” and interactive engagement. In this context then a thinking which is attuned to complexity and to otherness has an important place in the expression of the social subject as a complex and relational self (Seligman et al., 2008; Zeman, 2009; Di Paolo and De Jaegher, 2012; Han, 2017). These are not however social realities as we find them either in large-scale social schemata, or indeed in the intimacy of the face-to-face. As the concept of credition embraces processes in individuals as well as in groups or societies it is argued in this article that the concept of credition encourages a cohesive systematization of human behavior. In contrast with coherence in conventional science, “credition-centered” thinking finds expression in systemic ways while credition-centered learning might be best described as “the productive knowledge of community,” where community is generated by productive enhancement and the embrace of otherness over time (Gallagher, 2008; Tononi et al., 2016; Bente and Novotny, 2020).

Therefore this article intends to contribute to the “Architecture of Creditions” from a specific perspective which focusses on the combination of different poles, namely “Openness and Otherness.” From this perspective the terms “Openness” and “Otherness” together are understood to be key aspects for a definition of creditions. This article seeks to address a far-reaching problem concerning the nature of productive human relations. The academy is used to large scale population studies on the one hand and to small scale (face-to-face) sociality on the other. The intervening level of extended intimacy or productive sociality is far less present. And yet this is the level at which our “belonging” appears and is stabilized. It is the domain of our integrated identity. It is here then that the concept of “creditions” has a critical role to play. “Credition-theory” allows the emergence of “otherness” as a form of social openness. This in turn opens up to the sphere of ritual, in which the material properties of the linguistic sign, as shape and sound, are celebrated, in accordance with the presence of our advanced linguistic consciousness (Bell, 1992; Konvalinka and Roepstorff, 2012; Ramstead et al., 2016). Credition theory however enables the unfolding of a further analytical stage. This is the development of our understanding of the linguistic sign as mediating freedom. Credition theory can offer the realization of a typology of freedom, as a key factor in the development of our self-understanding, through the embrace of “openness” and “otherness” (Anderson, 2016). Integrating both concepts seems to be the basic challenge for learning the higher prosocial level.

## Beliefs as results of believing and believing processes

The credition concept highlights the dynamic of believing processes which result in mental representations which might be called beliefs. One of the innovative aspects of this approach results from neurophysiological findings which focus on specific believing processes (Seitz et al., 2018; Seitz and Angel, 2020). Three types can be distinguished. These are labeled as empirical, relational, and conceptual beliefs. These processes contribute in mutual interaction to the production of beliefs. My focus will be on conceptual beliefs. They are language-bound, narrativist and participative; and they involve ritual. This generates a stance of “believing in.” “[G]iven the involved neural processes of meaning-making and affective loading, conceptual beliefs appear similar to empirical and relational beliefs but are far more abstract” (Seitz and Angel, 2020, p. 3).

The capacity to develop more complex believing processes can be seen as the result of brain evolution. “The neural processes underlying formation and maintenance of beliefs in an increasingly complex social environment demanded augmented processing resources in the brain” (Fuentes, 2017; Seitz and Angel, 2020, p. 3–4). There is evidence for the possibility that “this enhanced processing demand was the force driving the phylogenetic enlargement of the parietal and frontal cortex which are key cortical areas in cerebral circuits affording integrative supramodal information processing” (Seitz and Angel, 2020, p. 3–4). The crucial further point here is that human complexity points to choice and so also to the complex phenomenon of freedom.

## Evolution, rituals, and tool use

Seitz and Angel propose that there is a consistent link between “conceptual beliefs” and “ritual,” whereby multi-modal complexity is constantly enhanced (Whitehouse, 2021). But what is the most concrete evolutionary source of this complexity? It has been proposed that the so-called “ratcheting effect” (Tennie et al., 2009) has played a key role whereby two different orientations in the world – interfacial orientation and hand-world tool use in combination – generated a new system which itself represents enhanced creativity. Advanced linguistic consciousness then is based in the interplay of the human interface and sociality on the one hand, and tool use or technology on the other. These are powerful, rotating, evolutionary drivers. From this perspective, words can be defined as “social tools” which combine sociality and technology in their original pre-modern setting.

Recent experiments in the learning of stone tool-making techniques reinforce the role of technology in the origins of language (Hurford, 2007; Lombao et al., 2017). Clark (2011) has pointed to the ways in which language and stone use mirror each other. In turn, Jayne Wilkins has argued for the emergence of “dialects” in key areas of the Still Bay and Howieson’s Port in Southern Africa, on the basis of “imitative social learning” and discrete sets of “stone tool technological traits.” Wilkins argues persuasively that distinctive sequences of strikes but also of the sounds of tool-making developed, and were expressed as distinct “dialects” or “schools” which paralleled the emergence of distinctive linguistic dialects (Stout and Chaminade, 2012; Wilkins, 2020; Dunbar, 2022).

But how are we shaped by this inheritance today? Firstly, according to Saussurean linguistics from the early 20th century, each utterance (parole) requires a choice between a range of potential linguistic possibilities (langue). We choose our words from all the available words we might have used, and so we allow ourselves to be held to account for them (De Saussure, 1986). Saussurean linguistics reinforces the role of freedom therefore, as arising from the internalization of external tools, through life-long practices of speaking and writing which together constitute our Advanced Linguistic Consciousness or “ALC” (Chalmers, 2010; Huth et al., 2016).

## Re-reading historical concepts with the modern lenses of cognitive science

It is increasingly evident today that there are no grounds for uncoupling our positivist, controlling freedom “from” and freedom “to” from the strongly consensual, rhythmic social modalities of our human “social cognition,” which is our freedom “in” (Schilbach et al., 2013; Bente and Novotny, 2020; Davies, 2021). Indeed, this broader integration arguably marks the point of a deeper humanization, and indeed is perhaps the locus of our power of choice. But we need to take note too of the effect of “otherness.” Creditions theory allows the coexistence of a community at Time “A” and Time “B.” Time “A” might be the launch of the Franciscan community in the 13th century with records of its compelling need to come to judgment about this new, enriched but also very challenging form of ethical life. Time “B” on the other hand may be the current reader’s own time framework. In Time “B,” those who have been influenced by contemporary community-based credition theory may well empathize with the records and data of Time “A.” It may be that Time “B” and Time “A” can interact with one another, as Time “B” discerns the “otherness” of Time “A” and begins openly to engage with it and to learn from it, in the formation of a trans-historical community based upon the reception of a productive “otherness.” The productivity of “creditions” needs to be grounded both in the cultural and the historical forms of our sociality, on the one hand, and in the contemporary science of human sociality, on the other.

Dante (1265–1321) offers the classical, transformational definition of language, which is that language is both sensuale and rationale. This means that “language, as a system of visible or oral signs, reproduces that peculiarly human mix that we ourselves are, of matter and mind, materiality and conceptuality,” reflecting the concept of “rational animality” as developed by Thomas Aquinas (Turner, 2004, p. 89–93; Davies, 2015, p. 248). In De vulgari eloquentia Dante writes: “It is more truly human for a human being to be perceived than to perceive” (Botterill, 1996, 1.3.7). The Divine Comedy is the cosmic enactment of that reality which is, as such, deeply consistent with the integrated science of our own times. Here Dante offers us a profound image of our “freedom in” on a cosmic scale which parallels current thinking on the role of the materiality of language in human relationality and human cognition.

The text known as the Summa Halensis (SH) was collaboratively authored by the founding members of the Franciscan school at Paris (1236–1245). It was not only the first official statement of Franciscan thought but also became a defining text which explored fundamental distinctions between philosophy and theology (Saccenti, 2020; Schumacher, 2020). It is this text, together with the later writings of the Franciscan scholar Duns Scotus, which appear to break new ground in understandings of the long-term practices of human sociality as manifest in “immersiveness” (4d i), “social cognition” (4d ii), “prosociality” (4d iii), and “symmetry” (4d iv) (see Table 1). (4d i–iv) can all be identified as modes of openness toward otherness. These are core representations of creditions as ways of integrating openness within complexity.

**TABLE 1:** Medieval and modern concepts of sociality.

Identifier	Original theme	Keyword	Modern application	Level of correspondence (1–5)
3d (2I)	“Thisness”	Haecceitas	Immersiveness	(4)
3d (2II)	“Natural Law”	Impressa	Social cognition	(5)
3d (2III)	“Projected sociality”	Condilectio	Prosociality	(5)
3d (2IV)	“Decision-making”	Non-velle	Symmetry	(4)

This table represents the comparison between early Franciscan notions of the social and current scientific conceptions of the social on a scale of 1–5. 1 represents minimal similarity and 5 represents extensive similarity.

Haecceitas – “immersiveness”

Scotus roots his anthropology in space and time and in our embodied human particularity. But he also develops an innovative metaphysics of particularity or what he calls haecceitas (“this-ness”). Haecceitas signals that we cannot define real things through the language either of “matter” or “form” alone, but neither can we define them through “matter and form” in combination, as was the norm. This also is too abstract. Rather, haecceitas points to real things as being a particular combination of both “matter” and “form” in this space and time. This finds parallels among contemporary philosophers today (e.g., Dancy, 2018). Scotus’ emphasis on particularity and “this-ness” yielded a new kind of metaphysics, one which participates, for Scotus, in the beauty of the original divine creation. This points to the otherness of the particular.

Impressa – “social cognition”

The SH argues decisively for an account of morality which is based in “natural law.” Once again the thinking is physicalist: “natural law is knowledge of the eternal law impressed in the soul.” Here our sense of morality is a given. The early Franciscans argued that “the eternal law is received by rational creatures and thus it is made present to their minds through impression rather than through an autonomous search on the part of reason itself.” (Saccenti, 2020, p. 227–250). This is arguably a physicalist ethics or an ethics of embodiment. It corresponds well with our own contemporary accounts of the role of the social cognition system as embedded, and as constituting the active ground of our social understanding and social bonding.

Condilectio – “prosociality”

The Franciscans were drawn in particular by the concept of condilectio as “shared love” or “co-love,” which they also understood to be related to a “love of justice.” As Lydia Schumacher states: “co-love occurs when a third is loved by the two in harmony and collectively (concorditer et socialiter) so that the two persons’ affects are fused to become one because of the flame of love for the third.” (Schumacher, 2019, p. 174). In its original context this is a version of Trinitarian theology, which places a particular emphasis on the “third” beyond the dyad of the “inter-face.” But we can also read this today as proposing “love” as a form of radical openness which is actualized beyond the “interface.” This appeals to the extension of love, as based in the social cognition system, into larger scale society, along the axis of a universalist “love for justice.”

Non-velle – “symmetry”

The Franciscan vocation itself (which involved a vow of radical poverty) focused the minds of leading intellectuals, and especially Duns Scotus, on the nature of decision-making. For Scotus, three kinds of freedom predominate: velle (“I want”), nolle (“I don’t want”) and non-velle (“my mind is still open”) (Ingham and Dreyer, 2004, p. 146–172). Velle and nolle both point to a form of self-interested possessiveness (affectio commodi), while the third points to our preparedness to remain detached and open in our moral questioning. Scotus calls this affectio iustitiae, or “love for justice.”

Here parallels emerge between openness in decision-making as Scotus and the early Franciscans develop it (Schumacher, 2020) and the neurological work, for instance, of Robert Kane. Kane describes how ethical challenges are represented in “movement away from thermal equilibrium – in short a kind of stirring up of chaos in the brain that makes it sensitive to micro-indeterminacies at the neuronal level.” Kane observes that the brain is a kind of “parallel processor (…) which can simultaneously process different kinds of information relevant to tasks such as perception or recognition through different neural pathways.” This processing capacity is “essential to the exercise of free will.” Kane adds that “[t]he key to difficult ethical decision-making, in which none of the initial possibilities appear to allow resolution, is time, effort and finally the formation of new neural pathways in the brain through the top-down effect.” These create the possibility of a new future and identity, and they constitute “growth” (Kane, 2011).

## Discussion

We can postulate that “creditions” can ultimately be defined in terms of the openness of the self as emergent within evolutionary contexts, involving “the phylogenetic enlargement of the parietal and frontal cortex which are key cortical areas in cerebral circuits affording integrative supramodal information processing” (Seitz and Angel, 2020, p. 3–4). The credition-centered thematization of complexity which is undertaken in the present project itself constitutes an openness to, or within, complexity. Furthermore, this openness bears the characteristics of freedom, or play, as an originary and fundamental characteristic of the human. Playing together is one of the key ways in which we develop and express our humanity. In particular, play can also be characterized in terms of freedom, or irrepressible non-compulsion.

It is this aspect of “freedom,” within an “architecture of complexity and otherness,” which begins to open up the possibility that the hermeneutics developed within creditions-theory may also overlap more directly with other forms of human self-possession; and specifically with that kind of self-possession which we can identify with the self’s belonging. Our social belonging is grounded in our acceptance by the other. The free movement of the other is prior. But this points to a further configuration, which is the foundational role of co-ordinated movement within relationality, as we speak and interact with each other, not least through maintaining eye contact. If they are viewed from another perspective and from within a different set of presuppositions, the spontaneity of such movements can be judged from the perspective of the terminology of ritual and repetition. Here it may appear that the freedom of movement we associate with the spontaneity of formal ritual can re-emerge as a form of life, and so contribute to a new phase in our human self-understanding.

## Author contributions

The author confirms being the sole contributor of this work and has approved it for publication.
